# Implementation of Time Temperature Indicators to Improve Temperature Monitoring and Support Dynamic Shelf Life in Meat Supply Chains

**DOI:** 10.1007/s41783-019-00080-x

**Published:** 2019-12-05

**Authors:** Antonia Albrecht, Rolf Ibald, Verena Raab, Werner Reichstein, Dietrich Haarer, Judith Kreyenschmidt

**Affiliations:** 1 Faculty of Agriculture, Institute of Animal Science, University of Bonn, Katzenburgweg 7-9, 53115 Bonn, Germany; 2 Faculty of Logistics Management, European University of Applied Sciences, Kaiserstraße 6, 50321 Brühl, Germany; 3 Department of Physics and BMIF, University of Bayreuth, Universitätsstr.30, 95440 Bayreuth, Germany

**Keywords:** Time temperature indicator, Temperature monitoring, Predictive modelling, Food waste, Remaining shelf life, Dynamic shelf life

## Abstract

Based on the well-investigated OnVu™ TTI kinetics, models were developed to adjust the label to different food products and predict the discolouration process under dynamic temperature conditions. After the successful validation under laboratory conditions, the applicability of the time temperature indicator (TTI) as shelf life indicator was tested in a national poultry chain. The TTI accurately reflected the temperature fluctuations occurring under real chain conditions. Shelf life predictions based on the discolouration of the TTIs were in accordance with the microbial shelf life of the product. The models were integrated in an online software tool to check for the compliance of the cold chain and predict the remaining shelf life of the product. The implementation of TTI and the software result in a valuable tool to support the decision-making process in the cold chain. The application of flexible shelf life enables the reduction of food waste in the meat supply chain.

## Introduction

Temperature monitoring in food chains is of vital importance for ensuring safe and high-quality products and reducing food waste [6, 32, 35, 40]. Until now, the temperature measurement in the chain is conducted by different methods and the control is mostly focused at the company level, but not over the entire supply chain. As a consequence, typical weak points may often stay undetected, but it is crucial for preserving food quality and safety [32, 38]. One way of controlling the temperature history of a product, from the packaging process to consumption, is the implementation of time temperature indicators. These labels continuously monitor and reflect the temperature history along the entire cold chain in a simple and cost-effective way [11, 21, 28, 43]. Several different TTI technologies have been studied under laboratory conditions and were published in the scientific literature [20, 22, 26, 41, 48]. Aside from the practical application of TTIs, the sophisticated task of implementation has been described as well [6, 9, 10, 44, 47]. According to these investigations, a proper reaction to changing temperature conditions and compliance with the spoilage kinetics of the product is crucial for the applicability of TTIs under practical conditions. Nevertheless, comprehensive pilot studies or persistent implementations in real distribution chains are rare. Even though the general applicability of TTIs to fundamentally improve cold chain management was reported, practical implementation often has been delayed by concerns about toxicity, missing efforts at the industry side, legislative questions or liability issues [8, 11, 19, 42]. But during the recent discussions about food waste, the high potential of intelligent packaging was spotlighted again [6, 40]. There is an urgent need to act, especially for sensitive products with a high environmental impact during production, such as meat [16, 35, 46]. Since a mismatch between the printed best-before date and the real status of the product was often reported [18], the implementation of TTIs offers the opportunity to monitor on single item level and integrate a dynamic shelf life for the product [5]. For a successful implementation into cold chain monitoring systems, several requirements have to be fulfilled. Next to an accurate monitoring of the temperature, an appropriate assessment of the remaining shelf life, indicated by the colour of the TTIs, is of vital importance for the delivery of safe, high-quality products and for the reduction of food waste. A further prerequisite for a successful implementation of TTIs as a shelf life monitoring device, besides the accordance between food and TTI kinetics, is a feasible implementation of the information delivered by the TTI in the decision-making process of the companies. Hence, models are required for the calculation of the particular warning and action control ranges at specific inspection points, based on the colour change of the label [36]. Such models have to be integrated in a user friendly software tool which simultaneously delivers information about corrective actions in case of temperature abuses [31, 33, 36]. Furthermore, the combination of logistics management systems, predictive quality models, and network architecture, such as wireless communication and cloud computing, can deliver an important contribution to the information flow and quality communication for all actors in the chain [6, 29, 31]. As a result, an efficient implementation of TTIs into process control is possible, which takes the discolouration of the TTI into account, as well as its link to the remaining shelf life of the monitored product. Until now, such models and software tools to support a dynamic shelf life are not available for meat supply chains.

Thus, the aim of the study was to validate the applicability of the photochromic OnVu™ TTI as an indirect freshness indicator for fresh poultry and to develop a TTI monitoring software tool to support the decision-making process in meat supply chains. Since this TTI is already well described and characterised, modelling and further developments of this study are based on the investigations of Kreyenschmidt et al. [26].

In the first step of the present study, an activation model was developed to adapt the discolouration process of the label to products with different shelf lives. A second model was developed to predict the discolouration of the label under dynamic temperature conditions. Both models were validated under laboratory conditions. In a third step, the applicability of the TTI as a freshness indicator and shelf life indicator for fresh poultry meat was tested under practical conditions. Based on the results, a software tool was developed (step four). The software can be used to support the decision-making process in companies by delivering information about the compliance of the cold chain and about the remaining shelf life of the product in each step of the chain.

## Materials and Methods

### Characteristic of the OnVu™ TTI Samples

The analysed photochromic TTI was the OnVu™ label B1 + 081126 (Ciba Specialty Chemicals & Freshpoint, Basel, Switzerland, patent WO ⁄ 2006 ⁄ 048412). The label, or rather the pigmented water ink, is activated by UV light, resulting in a blue colouring of the label. The discolouration of the label is characterised by a colour change from blue to white. The discolouration time and the kinetics of the label depend on the amount of light used for the charging process. During activation, the TTI can generally be adjusted to different products by varying the charging time, resulting in a modified intensity and duration of the light pulse.

For quantifying the colour change of the TTI and determining the shelf life of the label, the square value (SV, Eq. 1) of the TTI response was measured by a spectrophotometer (X-riteEyeOne i1^Basic; Gretag Macbeth, Regensdorf, Switzerland) using the CIELab colour system.

1 $${\text{SV}} = \sqrt {L^{2} + a^{2} + b^{2} } ,$$

where SV is the square value, *L* is the lightness, *a* is the red and green component, and *b* is the yellow and blue component of the label.

The colour change of the label or the development of the square value as function of time can be described by a logistic model [26]. The following equation was used to calculate the discolouration time (shelf life) of the TTI.

2 $${\text{SV}} = \frac{A}{{1 + e^{{ - k\left( {t - t_{\text{r}} } \right)}} }},$$

where SV is the square value of the TTI, *A* is the amplitude, *k* is the reaction rate (h^−1^), *t*_r_ is the reversal point (h), and *t* is the time (h).

The Arrhenius equation was used to describe the temperature dependency of the label discolouration [26]. Thus, the reaction rate can be plotted as a function of temperature.

3 $$\ln \left( k \right) = \ln \left( {k_{0} } \right) - \frac{{E_{\text{a}} }}{R} \cdot \frac{1}{T}$$

where *k* is the reaction rate (h^−1^), *k*_0_ is the constant (h^−1^), *E*_a_ is the activation energy (kJ mol^−1^), *R* is the ideal gas constant (8.314 J mol^−1^ K^−1^), and *T* is the absolute temperature (K).

A detailed characterisation of the discolouration process and the activation energy of the label calculated by the Arrhenius Model (23.22–25.77 kcal/mol) were given by [26, 36]. Data from these studies were the basis for the development of the following model and software tool.

### Modelling Approach

#### Development of an Activation Model to Adjust TTIs to the Product

In the first step, an activation model was developed to adjust the label to products with different shelf lives at a certain temperature. Initially, the model predicts the discolouration process of the TTI for several isothermal temperature conditions in relation to TTI activation. Based on the TTI discolouration time (shelf life) and storage temperature, the model calculates the appropriate charging and required initial SV of a label.

The influence of the initial SV (SV_0_) on the shelf life of the TTI was modelled by linear regression. Therefore, discolouration times for different temperatures were log transformed and modelled as a function of the SV_0_. The relation between discolouration time and SV_0_ was plotted for each investigated temperature (2–20 °C) and fitted to the following equation (Eq. 4):

4 $$\ln \left( {\text{SL}} \right) = a_{\text{act}} + b_{\text{act}} \cdot SV_{0} ,$$

where SL is the shelf life (discolouration time) of the TTI (h), *a*_act_ is the intercept, *b*_act_ is the slope of the linear fit (h^−1^), and SV_0_ is the initial SV of the TTI.

The estimated model parameters (intercept and slope) were used for further modelling. As discolouration shows a twofold dependency of SV_0_ and temperature [26], the slope (*b*_act_) and intercept (*a*_act_) were a function of temperature (Eqs. 5 and 6).

5 $$a_{\text{act}} = a_{\text{temp}} + b_{\text{temp}} \cdot T,$$

where *a*_act_ is the intercept of Eq. 4, *a*_temp_ is the intercept, *b*_temp_ is the slope of the linear fit, and *T* is the storage temperature.

6 $$b_{\text{act}} = a_{\text{temp2}} + b_{\text{temp2}} \cdot T,$$

where *b*_act_ is the slope of Eq. 4, *a*_temp2_ is the intercept, *b*_temp2_ is the slope of the linear fit, and *T* is the storage temperature.

After re-arranging Eq. 4, Eqs. 5 and 6 were inserted to calculate SV_0_ in relation to a specific storage temperature and shelf life (Eq. 7):

7 $${\text{SV}}_{0} = \frac{{\ln \left( {\text{SL}} \right) - \left( {a_{\text{temp}} + b_{\text{temp}} \cdot T} \right)}}{{\left( {a_{\text{temp2}} + b_{\text{temp2}} \cdot T} \right)}},$$

where SV_0_ is the charging time of the TTI, *T* is the storage temperature, SL is the discolouration time of the TTI (h), *a*_temp_ and *b*_temp_ are the intercept and slope of the temperature-dependent *a*_act_ (Eq. 4), and *a*_temp2_ and *b*_temp2_ are the intercept and slope of the temperature-dependent *b*_act_ (Eq. 4).

#### Development of a Dynamic TTI Model

To predict the discolouration of the TTI under fluctuating temperature conditions, a dynamic TTI model was developed. Based on the description of Kreyenschmidt et al. [27], the logistic equation (Eq. 2) and the Arrhenius equation (Eq. 3) were combined. Every temperature change was treated as a time interval and modelled separately. Using this method, the logistics equation predicts the discolouration with time, while the Arrhenius equation describes the temperature dependency. The temperature dependency of the variable A was modelled by linear regression. At the end of every particular interval, the *SV* was computed as follows (Eq. 8):

8 $${\text{SV}}_{e} = \frac{{A_{\text{T}} }}{{1 + e^{{ - k_{\text{T}} \left( {t - t_{{{\text{re}} - 1}} } \right)}} }},$$

where SV_*e*_ is the response of the TTI at the end of the particular interval, *e* = 1…*n* is the  number of intervals, *A*_T_ is the temperature-specific amplitude of the interval, *k*_T_ is the temperature-specific reaction rate of the interval (h − 1), *t*_r_ is the reversal point (h), and *t* is the time (h).

For every interval, the reversal point was calculated with the particular values of *A* and *k.* Thereby, the SV of the previous interval was applied (Eq. 9).

9 $$t_{\text{r}} = \frac{{\ln \left( {\frac{{A_{\text{T}} }}{{{\text{SV}}_{e - 1} }} - 1} \right)}}{{k_{\text{T}} }} + t,$$

where SV_*e* − 1_ is the TTI response at the end of the former interval, *e* = 1…*n* is the number of intervals, *A*_T_ is the temperature-specific amplitude of the interval, *k*_T_ is the temperature-specific reaction rate of the interval (h − 1), *t*_r_ is the reversal point (h), and *t* is the time (h).

For the first interval *n*(*t*) = 1, the SV_0_ is inserted. By executing the TTI kinetics model, the discolouration process of the TTI can be modelled under dynamic temperature conditions, resulting in an exact prediction of the SV for any point in the cold chain. Thus, the model enables the calculation of check values and warning ranges for specific temperature limits at inspection and control points.

### Model Validation Under Laboratory Conditions

The validation of the TTI model was conducted with 120 TTIs which were positioned on the top, the four sides, and the bottom of two cardboard boxes. Each box (40 cm × 30 cm × 10 cm) was filled with 5 kg of poultry filet wrapped in polyethylene foil to simulate the delivery unit in the supply chain. One box with 60 TTIs was stored under fluctuating temperature conditions simulating the temperature profile of a German poultry chain [37]: 24 h at 4 °C, 2 h at 5 °C, 6 h at 1 °C, 2 h at 5 °C, 12 h at 2 °C, ending with 4 °C until the end of shelf life. The other box, also with 60 labels, was stored at a constant temperature of 4 °C (mandatory storage temperature according to EU directive 853). Additionally, every side was monitored by data loggers recording the temperature every 5 min with a margin of error of ± 0.5 °C (Verdict-Systems BV, Aalten, Niederlande).

Based on the product characteristics (Activation energy = 24.6 kcal/mol, shelf life = 140 h) described by Bruckner et al. [3], the TTI was activated with UV light to an SV_0_ of 57.9. This value was calculated with the TTI activation model described under 2.2.

After activation of the label, the boxes were stored in high precision low temperature incubators (Sanyo Electric Co., Ora-Gun, Gunma, Japan). The discolouration process was monitored every 24 h by measuring the SV of the TTIs as described in Sect. 2.1. Further on, temperature recordings of the data loggers were used to model the TTI response. A comparison of measured and predicted SV was applied to validate the TTI model. The model performance was estimated using the coefficient of determination *R*^2^, the bias factor, and the accuracy factor [39].

### Validation of the TTI as a Shelf Life Indicator in a Real Supply Chain

For assessing the suitability of the TTI as a shelf life indicator for fresh poultry meat, the TTI was tested under practical conditions. The field trial was conducted in a German poultry chain and comprised the monitoring of eight cardboard boxes in 4 weeks, containing 5 kg of poultry filet in each case. The chicken breast was packed aerobically in the boxes and wrapped in transparent foil.

Due to improvements of hygienic conditions in the production plant [3], the shelf life of the poultry filets was extended to 168 h at 4 °C during the field trial. Thus, the TTI tags were activated to an initial SV of 57.4 for an appropriate match between the label and the food product. TTIs and data loggers were placed as described for the laboratory investigations, resulting in a total of 480 activated TTIs for the eight cardboard boxes.

The boxes were distributed through the typical German poultry chain (producer, transport, wholesaler, transport, butcher). After the last transportation step (butcher), each cardboard box was stored until the end of its shelf life at 4 °C in a cooling chamber in the laboratory (Viessmann CS 1300 T-111, Viessmann Werke GmbH & Co KG, Allendorf, Germany). During the study, the storage temperature at the boxes was in the range of − 1 °C to + 12 °C and maximum temperatures between + 5 and + 15 °C.

Measurements of the TTIs response were applied at several inspection and decision points in the chain, resulting in a complete monitoring of the whole cold chain. Additionally, microbial samples were taken at seven points (0 h, 24 h, 72 h, 96 h, 168 h, 192 h, 216 h) to investigate the applicability of TTIs as shelf life indicator. At each microbial inspection point, three poultry samples were taken per box (in total *n* = 168). Since *Pseudomonas* spp. is the specific spoilage organism (SSO) for aerobe-packed fresh poultry filet, investigations focused on this organism. A representative sample of 25 g of the meat was transferred to a stomacher bag. After adding 225 g chilled saline peptone diluents (0.85% NaCl with 0.1% peptone; Oxoid, Basingstoke, United Kingdom), the sample was homogenised for 60 s in a Stomacher 400 (Kleinfeld Labortechnik, Gehrden, Germany). An appropriate 10-fold dilution of the homogenate was prepared using saline peptone diluents. The suspension was tested for *Pseudomonas* spp. by surface spreading on *Pseudomonas* Agar Base (Oxoid Basingstoke, United Kingdom) plus CFC supplement (Oxoid, Basingstoke, United Kingdom). After incubating the petri dishes aerobically at 25 ± 1 °C for 48 h, the microbial counts were determined. The product was classified as spoiled when *Pseudomonas* spp. reached a level of 7.5 log_10_ cfu/g.

The discolouration process of the TTI was compared with the microbial growth during storage to assess the applicability of the TTI as a shelf life indicator.

### Development of a Software Tool to Support the Decision-Making Process in Meat Companies

Based on the previous results and the developed models (2.1–2.4), an online Software tool was developed to support the decision-making process in cold chains using TTIs. By integrating the activation and dynamic models, the TTI can be adjusted to specific products to calculate if the cold chain was in its limit. The software delivers information about the temperature history and the remaining shelf life of the product based on the measured TTI value. The tool also allows the definition of specific warning and action ranges for the incoming and outgoing inspection. For the software development, the main goal was to separate modelling from content management system, achieve portability and use open source tools exclusively. A main focus was the easy maintenance and ability for further developments. The online tool is embedded into a wordpress system. The backend programming was conducted with PHP and the frontend programming is a generated javascript (via coffeescript). Data exchange between frontend and backend is conducted via AJAX. A MySQL database was set up with a wordpress interface. Thus, flexible adjustments are possible, if discolouration kinetics of the TTI change with new developments or enhanced prototypes. Different TTI systems or food products can easily be added to the database. Thus, the tool can be extended to a variety of TTI systems and food products, and act as a universal tool to support cold chain management.

## Results and Discussion

### Validation of the Developed Models Under Laboratory Scale

**Fig. 1:**
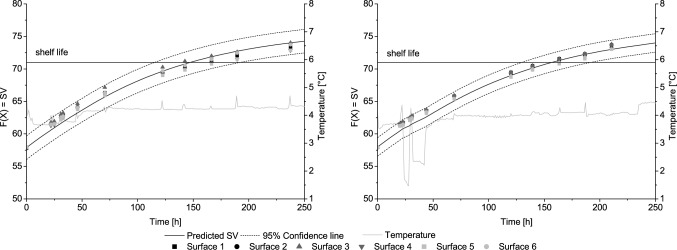
Observed (data points) and predicted response of the TTI under isothermal temperature conditions on cardboard box 1 (left) and under non-isothermal temperature conditions of 4 °C on cardboard box 2 (right)

After storing the labels under dynamic and constant temperature conditions, the measured TTI values were compared to the predicted TTI response. As shown in Fig. 1, there was a high agreement between measured and predicted values.

The dynamic TTI model generally showed a very good performance, as the *R*^2^ was above 0.96 on all sides of box 1 and 2 (Table 1). The bias factor ranged between 0.98 and 1.00 and the accuracy factor ranged between 1.00 and 1.01 for both scenarios. Based on the indices for performance evaluation, the model showed a nearly perfect agreement between predicted and observed values [39]. The differences between predicted and observed TTI shelf life at the single sides of the boxes varied between 2 and 18 h (control) and 1 and 22 h (dynamic), respectively. The maximum aberrations of 18 h and 21 h were observed at the TTIs placed on the side of the boxes. These aberrations are probably caused by air flows arising during cooling cycles of the incubator. Very low aberrations (7 h at control and dynamic scenario) were observed at the bottom of the boxes.

**Table 1:** Model performance and shelf life prediction of all TTIs during laboratory validation

	Mean SL observed (h)	Mean SL predicted (h)	Mean deviance (h)	Mean Bf	Mean Af	Mean *R*^2^
Control	159	150	13	0.99	1.01	0.98
Dynamic	157	157	11	0.99	1.01	0.98

*SL* shelf life, *Bf* bias factor, *Af* accuracy factor, *R*^2^ coefficient of determination

In conclusion, the laboratory validation showed that the activation model provides the information for the adjustment of the TTI on product characteristics. Based on the calculated activation time, the TTI was charged suitably for a product shelf life of 144 h at 4 °C. Additionally, the dynamic TTI model reliably predicted the discolouration of the TTI reacting to the simulated temperature profile during model validation in the laboratory. Thus, the TTI, as well as the model, is able to accurately reflect dynamic temperature conditions, which are important prerequisites for a successful implementation in real supply chains [14]. A reliable adjustment of this TTI and applicability as freshness indicator was also shown for seafood [2, 15, 30] which emphasises the flexibility of the OnVu™ TTI. A further prerequisite for a successful implementation into practice is a high reproducibility of the label under laboratory as well as real chain conditions, which was shown before by Kreyenschmidt et al. [26].

### Field Application of the Time–Temperature Indicator in a Poultry Supply Chain

The charging process under practical conditions showed a high reproducibility. The SV_0_ showed a standard deviation of 0.03 for all labels (*n* = 480). As stated by Kreyenschmidt et al. [26], constant environmental temperature conditions, which were observed during the pilot study, are mandatory during the charging process.

In practice, the TTI model displayed a very accurate prediction of the discolouration process of the label for all investigated boxes and the different sides of the boxes. This was reflected by high goodness of fit values (Bf = 0.99, AF = 1.01, *R*^2^ > 0.95). During the field trial, the TTI discolouration reflected the logged temperature accurately. The mean shelf life of the TTIs was varying for the different boxes between 174 and 192 h. These differences were caused by different placements of the investigated boxes in pallets and their location in the truck. The cyclical cooling system, which generates airflows between pallets and over the surfaces of the boxes for example, can result in different temperature conditions for the single units [17, 37]. These were reflected by the labels, and extended or shortened the discolouration times.

**Fig. 2:**
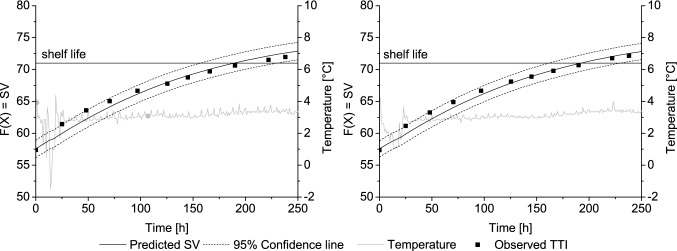
Measured SV of TTIs and predicted response during field trial at cardboard box 5: top of the box (left) and bottom of the box (right)

The temperature recorded during storage, as well as the measured and predicted square values for a typical box, is shown in Fig. 2. The results lead to the conclusion that the TTI and the dynamic model can be used as valuable tools for temperature control at vital inspection and decision points within the particular distribution chain. Thus, control values for the incoming and outgoing inspection can be calculated with the help of the dynamic TTI model. A calculation of warning ranges based on the very specific circumstances and needs of a certain supply chain can be determined as well with this model.

### Reliability of the TTI as Indirect Freshness Indicator

**Fig. 3:**
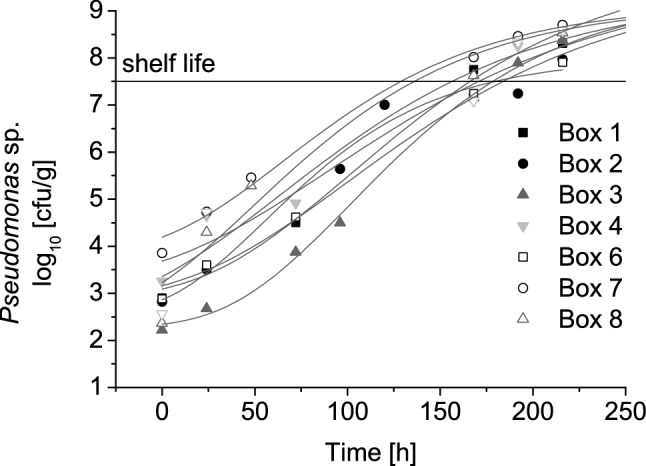
Growth of *Pseudomonas* spp. on poultry meat in each cardboard box with the respective fit using the Gompertz model

The TTI discolouration process was compared to the microbial growth to investigate the functional capability of the label as an indirect freshness indicator. The specific spoilage organism *Pseudomonas* spp. showed a variation of initial bacterial count (N_0_) between 2.28 to 3.86 log_10_ cfu/g at the beginning of storage. The development of *Pseudomonas* spp. in chicken breast filet during transportation and storage is shown in Fig. 3. The microbial growth shows the typical sigmoidal curve with maximum values of microbial load between 8.0 and 10.2 log_10_ cfu/g (Table 2). The average microbial shelf life evaluated by *Pseudomonas* spp. in all boxes was calculated as 162 h (SD = 20.55). The estimated mean shelf life is in accordance with the shelf life given by the producer and corresponds to the shelf life of aerobe-packed poultry estimated in previous studies [1, 4, 37].

**Table 2:** Parameters obtained from the Gompertz model for the different cardboard boxes

	N_0_ [log_10_ cfu/g]	N_max_ [log_10_ cfu/g]	µ_max_ (1/h)	R^2^	Microbial SL (h)	TTI SL Bottom (h)	Mean product temperature [°C] (SD)
Box 1	2.90	10.17	0.03	0.99	166.15	184	2.87 (0.27)
Box 2	2.55	8.00	0.04	0.95	179.52	177	3.59 (0.18)
Box 3	2.28	9.40	0.04	0.99	178.31	187	2.63 (0.45)
Box 4	3.26	9.16	0.03	0.95	170.70	184	3.16 (0.41)
Box 5	2.88	10.20	0.03	0.99	183.81	192	3.20 (0.44)
Box 6	3.86	9.15	0.03	0.99	128.72	173	3.26 (0.47)
Box 7	2.37	9.87	0.03	0.91	136.02	177	3.24 (0.34)
Box 8	2.56	9.80	0.03	0.93	156.29	173	3.75 (0.55)

*N*_*0*_, initial bacterial count; *N*_*max*_, maximum population level; *μ*_*max*_, maximum growth rate; *SL*, shelf life, *SD*, standard deviation

Growth parameters of *Pseudomonas* spp. on fresh chicken breast filet obtained with the Gompertz model are listed in Table 2. However, microbial shelf life varied between 129 h (box 6) and 184 h (box 5). A shortened microbiological shelf life for cardboard box 6 was caused by the high initial bacterial count of *Pseudomonas* spp. The remaining shelf life of products can be affected significantly by varying initial microbial loads [3]. Thus, highly controlled processes and a stable product quality at the beginning of the chain are crucial for the reliable application of TTIs as shelf life indicators [28]. Further on, an implementation of rapid detection methods would improve knowledge on the real status of the product in the early stages of the chain and thus support the accuracy of model predictions.

**Fig. 4:**
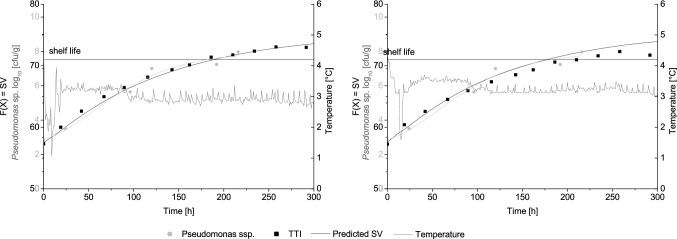
Representative temperature, TTI response and growth of *Pseudomonas sp.* shown for cardboard box 2. Top of the box (left) and bottom of the box (right)

Aside from box 6, the shelf life predictions based on the TTI discolourations are in good accordance with the shelf life estimated by the microbiological investigations (Table 2). The predicted and measured SV (labelled at the top and bottom) in comparison to the growth of *Pseudomonas* spp. on poultry is shown in Fig. 4 for cardboard box 2. In general, a mismatch less than 24 h between microbial and TTI shelf life is considered as acceptable [45] and was achieved with most boxes. Generally, the TTI slightly overestimates microbial shelf life of poultry. Since an overestimation classifies the product as consumable although the shelf life is already exceeded, it leads to risks to the quality and the safety of the product [13, 28]. However, the deviation between microbial and TTI shelf life was below 24 h and the integration of a safety margin can reduce the quality and safety risk. Temperature fluctuates within the storage, transportation units and over pallets, and thus influences the shelf life of the product [17, 18]. The printed best-before date was reported to show significant discrepancies to the real shelf life of the product [17, 18]. Thus, a monitoring on single item level is mandatory to establish dynamic shelf lives for fresh meat products [5, 6].

### Applying Software to Support the Decision-Making Process of Companies

The developed TTI predictor addresses to all actors handling the product and allows to calculate dynamic shelf lives and support the decision-making process in supply chain. The TTI predictor is available for free on https://ccm-network.com/tti-predictor-2/. By entering the product’s shelf life at a certain reference temperature, the initial SV value of the label can be calculated. To use the TTI as a shelf life control instrument, the user can measure the values of the TTI with a spectrophotometer in each step of the chain and integrate the value. To calculate the remaining shelf life, the date of packaging or the time since packaging has to be inserted. By comparing the measured SV of the TTI with the SV calculated by the model at the optimal storage temperature, the tool gives an output if the cold chain has been violated or is in its limit. Based on the measured TTI value, a calculation of the mean storage temperature occurred in the supply chain is given. Additionally, the remaining shelf life is calculated based on the optimal storage temperature for the product chosen in the first step.

After implementing the TTI system into the supply chain, monitoring of the SV is crucial at inspection points, to avoid shelf life losses and deliver products meeting the minimum acceptance level of the customer. The definition of warning and control ranges for specific inspection points enables a comparison between an ideal TTI response and the current response in the chain. As a result, the software displays a traffic signal (green, yellow or red), showing if there was a problem with the cold chain and, consequently, with the shelf life: A green signal is shown when the measured SV is smaller than the computed SV, whereas a red signal is shown if the measured SV is higher than the computed SV. Thus, the green sign corresponds to a cold chain where the temperature was within its limits and the red sign shows temperature abuse affecting the shelf life of the product. The specific warning and control ranges for the SV or calculated mean temperature can be set flexible in the database. Thus, the tool is easily adaptable to the specific needs at inspection points of the particular cold chain.

If the tool recognises that the cold chain was interrupted, a further option will open. The software is able to predict if it is possible to save the product by adjusting the cooling conditions. For meat products, adjusting the storage temperature is an efficient way to reduce food waste [7]. By utilising this information, the storage management of the company can be adapted to LSFO (least shelf life—first out) instead of FIFO (first in—first out). Additionally, logistics processes can be adapted so that products will be delivered to customer with shorter transport routes [29]. The accurate prediction of the remaining shelf life at every step of the chain enables the establishment of a dynamic shelf life for the product which can remarkably reduce food waste, especially when it is combined with discounting [5].

After the establishment of overall temperature monitoring along the distribution chain, the efficiency of information management has a similarly high impact on the success of the application of novel methods [12, 23–25, 34]. The successful operation of TTIs under practical conditions provides extended advantages when they are combined with sophisticated models [15, 29, 47]. The incorporation into user friendly software supports the implementation process and decision making during supply chain monitoring [33, 36]. However, a prerequisite for the successful implementation of such a software tool is a stable hygienic process, data exchange, and a close cooperation between the different participants of the chain. In addition, a reliable link between temperature data, quality indicators and the product’s characteristics is mandatory for a precise interpretation of the data [38]. Thus, the activation energy of the TTI has to fit the product’s activation energy [45]. Further recommendations can be generated on the basis of the outcome of the inter-organisational inspection scheme. These connections emphasise the complexity of optimising cold chain management using innovative temperature monitoring systems.

## Conclusion and Future Prospects

The TTI field trial and the validated TTI models showed that the TTI can support valuable management decisions in perishable food supply chains. As such, TTIs can deliver an important contribution to the monitoring of the shelf life of perishable products, and to the reduction of food waste. However, different technical, functional, and organisational requirements have to be assured prior to its integration within the supply chain.

Generally, the study has shown that the TTIs, as well as the TTI kinetics model, are a useable and useful tool for temperature monitoring during processing and for the prediction of remaining shelf life in different steps of the supply chain. Furthermore, the high model performance and consistency in the TTIs discolouration process in this study are the basis for a successful implementation in a real cold chain and establishment of dynamic shelf life. TTI predictions of remaining shelf life were very satisfying in the present pilot study. The software should be extended for smartphone application. A colour measurement of the TTI directly with smartphones is desired for an improved work flow and dynamic shelf life calculation online. During implementation, a detailed adjustment to the particular needs of the actors in the cold chain is required.
